# Gain-of-Function Mutation of Tristetraprolin Impairs Negative Feedback Control of Macrophages *In Vitro* yet Has Overwhelmingly Anti-Inflammatory Consequences *In Vivo*

**DOI:** 10.1128/MCB.00536-16

**Published:** 2017-05-16

**Authors:** John D. O'Neil, Ewan A. Ross, Michael L. Ridley, Qize Ding, Tina Tang, Dalya R. Rosner, Thomas Crowley, Deepak Malhi, Jonathan L. Dean, Tim Smallie, Christopher D. Buckley, Andrew R. Clark

**Affiliations:** a Institute of Inflammation and Ageing, College of Medical and Dental Sciences, University of Birmingham, Birmingham, United Kingdom; b Faculty of Medicine, Imperial College London, London, United Kingdom; c Kennedy Institute of Rheumatology, Nuffield Department of Orthopaedics, Rheumatology and Musculoskeletal Sciences, Oxford University, Oxford, United Kingdom

**Keywords:** dual-specificity phosphatase 1, inflammation, macrophages, posttranscriptional RNA-binding proteins, tristetraprolin

## Abstract

The mRNA-destabilizing factor tristetraprolin (TTP) binds in a sequence-specific manner to the 3′ untranslated regions of many proinflammatory mRNAs and recruits complexes of nucleases to promote rapid mRNA turnover. Mice lacking TTP develop a severe, spontaneous inflammatory syndrome characterized by the overexpression of tumor necrosis factor and other inflammatory mediators. However, TTP also employs the same mechanism to inhibit the expression of the potent anti-inflammatory cytokine interleukin 10 (IL-10). Perturbation of TTP function may therefore have mixed effects on inflammatory responses, either increasing or decreasing the expression of proinflammatory factors via direct or indirect mechanisms. We recently described a knock-in mouse strain in which the substitution of 2 amino acids of the endogenous TTP protein renders it constitutively active as an mRNA-destabilizing factor. Here we investigate the impact on the IL-10-mediated anti-inflammatory response. It is shown that the gain-of-function mutation of TTP impairs IL-10-mediated negative feedback control of macrophage function *in vitro*. However, the *in vivo* effects of TTP mutation are uniformly anti-inflammatory despite the decreased expression of IL-10.

## INTRODUCTION

Macrophages are at the forefront of innate immune-mediated defenses against infectious pathogens. They are equipped with several pattern recognition receptors (PRRs) with which they detect pathogen-associated molecular patterns (PAMPs) common to different classes of microbes, for example, lipopolysaccharide (LPS), a component of the cell wall of Gram-negative bacteria (1, 2). The engagement of macrophage PRRs initiates very rapid changes of gene expression in order to mobilize antimicrobial effectors, recruit and/or activate other cells of the innate and acquired immune systems, amplify danger signals, and coordinate effective cellular responses. Excessive responses of macrophages to the engagement of PRRs is potentially harmful, in the worst case leading to systemic organ failure and death. It is therefore unsurprising that macrophage activation by PAMPs is regulated by complex networks of positive and negative feedback mechanisms that together ensure rapid but proportional and temporally limited proinflammatory responses (3–7).

One important feedback mechanism involves the secretion of the anti-inflammatory cytokine interleukin 10 (IL-10). Particularly in tissues like those of the gut, which are continuously exposed to PAMPs, IL-10 is essential for restraining inflammatory responses to commensal microorganisms (8, 9). One critical function of IL-10 is the suppression of the *Il12b* gene, which codes for the shared p40 subunit of the cytokines IL-12 and IL-23. The constraint of IL-12 and IL-23 expression is required to prevent excessive Th1- and Th17-mediated intestinal inflammation (10–13). Macrophage-specific knockout of the IL-10 receptor (IL-10R) results in severe spontaneous gut inflammation (14, 15). Similarly, polymorphisms in the human IL-10–IL-10R axis are associated with susceptibility to inflammatory bowel disease (16). The anti-inflammatory actions of IL-10 are mediated by the transcription factor STAT3 (signal transducer and activator of transcription 3), which is phosphorylated and activated by the tyrosine kinases JAK1 and TYK2 upon the engagement of the IL-10 receptor. A number of STAT3-regulated target genes have been implicated as mediators of anti-inflammatory functions of IL-10 (6, 17–19). The other side of the coin is that increased expression of IL-10 may contribute to pathogenesis, for example, by impairing antitumor immunity (20). Several viruses express IL-10 orthologues, apparently as a means of evasion of the innate immune system (21). The expression of endogenous IL-10 is tightly regulated by different mechanisms in several cell types that can express this anti-inflammatory cytokine (22, 23).

Much is known about how transcription factors regulate the complex programs of gene expression in activated macrophages (3, 24–27). It is often overlooked that the dynamic regulation of mRNA stability also plays an important role (28, 29). Many inflammatory mediator mRNAs have adenosine/uridine-rich elements (AREs) in their 3′ untranslated regions, which act as binding sites for various RNA-destabilizing factors, the best characterized of which is tristetraprolin (TTP) (30, 31). TTP promotes the rapid turnover of its substrate transcripts by recruiting a number of nuclease complexes, notably the CCR4/NOT complex, which mediates poly(A) tail shortening, the rate-limiting step in the degradation of most mRNA species. In resting macrophages, TTP is expressed at very low levels. Its upregulation in response to proinflammatory stimuli constitutes a negative feedback mechanism to limit the duration and strength of inflammatory gene expression (30–32). Myeloid cell-specific disruption of the TTP gene (formally known as *Zfp36*) causes extreme sensitivity to lethal endotoxic shock, although there is no evident phenotype in the absence of inflammatory challenge (33, 34). In contrast, global disruption of the *Zfp36* gene causes a spontaneous and severe inflammatory syndrome with some features of rheumatoid arthritis (35). *Tnf* was the first mRNA shown to be posttranscriptionally regulated by TTP, and dysregulation of tumor necrosis factor (TNF) expression plays an important role in both myeloid cell-specific and global *Zfp36* knockout phenotypes (33–36). Nevertheless, it is clear that very many additional targets exist and are dysregulated in cells lacking TTP (30, 37, 38). Puzzlingly, IL-10 was identified as a target of posttranscriptional regulation by TTP, which was overexpressed by *Zfp36*^−/−^ macrophages (38–41). Enhanced IL-10-mediated negative feedback in *Zfp36*^−/−^ cells resulted in the paradoxical underexpression of IL-6, IL-12, and IL-23 (39, 41).

The mitogen-activated protein kinase (MAPK) p38 signaling pathway controls the expression of inflammatory mediators via the phosphorylation and inactivation of TTP (42, 43). Hence, MAPK p38 inhibitors decrease the expression levels of several proinflammatory mediators and destabilize the corresponding mRNAs in *Zfp36*^+/+^ but not *Zfp36*^−/−^ macrophages (41, 44). The critical phosphorylations, at serine 52 and serine 178 of murine TTP, are carried out by MAPK-activated protein kinase 2 (MK2), which is downstream of the MAPK p38 itself (43, 45–47). They result in the recruitment of 14-3-3 proteins, the disruption of the interaction between TTP and the CCR4/NOT complex, and the consequent protection of the target transcript's poly(A) tail (46, 48–50). The coupling between MAPK p38 signaling and TTP activity contributes to the precise timing of gene expression in activated macrophages (33, 38). Prolonged MAPK p38 activation in macrophages lacking the MAPK phosphatase dual-specificity phosphatase 1 (DUSP1) promotes the overexpression of several inflammatory mediators *in vitro* and *in vivo*, causing exaggerated responses to experimental challenges such as LPS injection (5, 51). To a large extent, this dysregulated inflammatory response is caused by the increased phosphorylation and inactivation of TTP, the increased stability of proinflammatory mRNAs, and a delay to the off phase of gene expression (52).

We recently used homologous recombination to generate a knock-in mouse strain in which Ser52 and Ser178 of the endogenous TTP protein are replaced with nonphosphorylatable alanine residues (53). The mutated *Zfp36* locus is known as *Zfp36*^*aa*^, and its protein product is known as TTP^aa^. Since it cannot be inactivated by the MAPK p38 pathway, TTP^aa^ is a constitutive, dominant mRNA-destabilizing protein. Its expression protects mice from harmful consequences of systemic LPS injection (53), experimental inflammatory arthritis (54), and inflammatory lung pathologies (43). Differential gene expression was assessed by microarray analysis of *Zfp36*^*aa/aa*^ macrophages, which express the alanine-substituted mutant form of TTP from the endogenous *Zfp36* locus (52, 53). Several known TTP target mRNAs, such as *F3*, *Tnf*, *Cxcl1*, *Cxcl2*, *Ier3*, and *Il10*, were underexpressed by *Zfp36*^*aa/aa*^ macrophages, and some putative novel TTP targets were identified.

We and others have discussed the possibility of therapeutic targeting of TTP in order to exert anti-inflammatory effects (31, 43). A proof of principle was generated by the treatment of experimental arthritis using two compounds that promote PP2A-mediated dephosphorylation and activation of TTP (54). A significant concern is that altering TTP function could give rise to unwanted proinflammatory effects by disrupting endogenous negative feedback mechanisms, for example, those involving IL-10 (37, 39–41). Here we investigated *in vitro* and *in vivo* consequences of the impaired expression of IL-10 caused by a gain-of-function mutation of TTP.

## RESULTS

### Anomalous expression of certain TTP targets by *Zfp36*^*aa/aa*^ macrophages.

**FIG 1:**
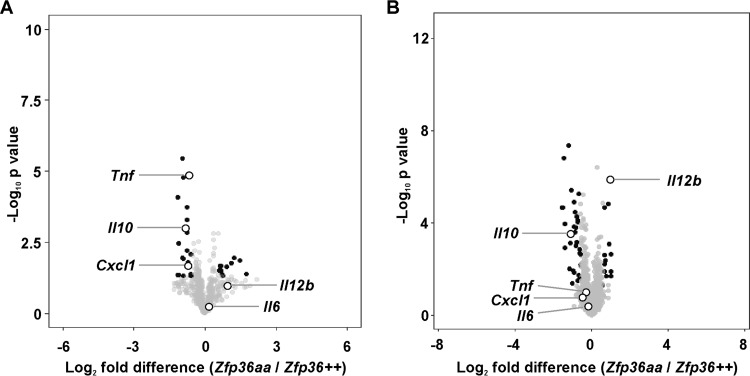
Differentially expressed transcripts in *Zfp36*^*aa/aa*^ M-BMMs. M-BMMs were generated from 3 *Zfp36*^+/+^ and 3 *Zfp36*^*aa/aa*^ mice and treated with 10 ng/ml LPS for 1 h (A) or 4 h (B). RNA was isolated, and transcript abundance was analyzed by using Agilent microarrays and Partek Genomics Suite. Transcripts expressed above an arbitrary threshold of 200 RMA in at least two replicates and demonstrating upregulation by LPS (>2 times; *P* < 0.05) were selected for display. Data are illustrated in the form of volcano plots, in which transcripts with a >1.5-fold difference in expression levels between *Zfp36*^+/+^ and *Zfp36*^*aa/aa*^ M-BMMs and a corrected *P* value of <0.05 are shaded black. Underexpressed transcripts are to the left of the origin. Several transcripts are highlighted.

Microarray analysis was performed to identify transcripts differentially expressed by *Zfp36*^*aa/aa*^ macrophage colony-stimulating factor (M-CSF)-differentiated bone marrow-derived macrophages (M-BMMs) after stimulation with LPS. The microarray data were deposited at the Gene Expression Omnibus, and some aspects of their analysis were previously reported (52, 53). Figure 1 illustrates the analysis of LPS-induced transcripts (>2-fold increase of expression in response to LPS; *P* < 0.05). The data are presented in the form of volcano plots, in which transcripts with a >1.5-fold difference of expression between *Zfp36*^+/+^ and *Zfp36*^*aa/aa*^ M-BMMs and a corrected *P* value of <0.05 are shaded black. Differential expression data are summarized in Table S1 in the supplemental material. As expected for a dominant negative regulator of gene expression, many transcripts were underexpressed by *Zfp36*^*aa/aa*^ M-BMMs. Several of the changes of expression at 1 h were previously reported, including the underexpression of *Tnf*, *Il10*, and *Cxcl1* mRNAs (53). By 4 h after the addition of LPS, the levels of both *Tnf* and *Cxcl1* mRNAs had almost returned to basal levels, and differences between *Zfp36*^+/+^ and *Zfp36*^*aa/aa*^ M-BMMs were no longer statistically significant (also see Fig. 4A). *Il10* mRNA was significantly underexpressed by *Zfp36*^*aa/aa*^ M-BMMs at both the 1-h and 4-h time points. Contrary to expectation, *Il6*, which was previously characterized as a TTP target (38, 41, 55, 56), was not significantly underexpressed by *Zfp36*^*aa/aa*^ M-BMMs (Fig. 1). Several other transcripts were significantly overexpressed by *Zfp36*^*aa/aa*^ M-BMMs. One example, *Il12b*, is highlighted in Fig. 1.

Gene ontology (GO) analysis was performed on transcripts differentially expressed by *Zfp36*^*aa/aa*^ M-BMMs (see Table S2 in the supplemental material). At both 1 h and 4 h, underexpressed transcripts were highly enriched in several GO terms relating to response to stimulus, defense response, or inflammatory response. The enrichment of these terms was a consequence of the differential expression levels of transcripts including *Tnf*, *Cxcl1*, *Cxcl2*, *Clcf1*, *Ier3*, *S100a8*, *Zfp36*, *Mefv*, *Ltf*, and *Il10*, most of which were previously described as TTP targets (30). These results are arguably trivial, given that LPS-induced transcripts were selected prior to the analysis of differential gene expression. GO analysis of the overexpressed transcripts was more informative. At 1 h, several GO terms related to cell migration were very significantly enriched (*P* < 10^−10^). All of these enrichments arose from the increased expression levels of four transcripts, namely, *Cited2*, *Cxcl10*, *Sdc4*, and *Trib1*. At 4 h, the most significantly enriched GO terms were related to the phosphorylation of the transcription factor STAT5 (signal transducer and activator of transcription 5) or the regulation of the extrinsic apoptosis pathway (Table S2). The corresponding lists of differentially expressed transcripts were dominated by *Il12b*, *Csf2*, *Cx3cl1*, *Kdr*, and *Rasip1*. These findings suggest that the genes overexpressed by *Zfp36*^*aa/aa*^ M-BMMs are functionally distinct.

**FIG 2:**
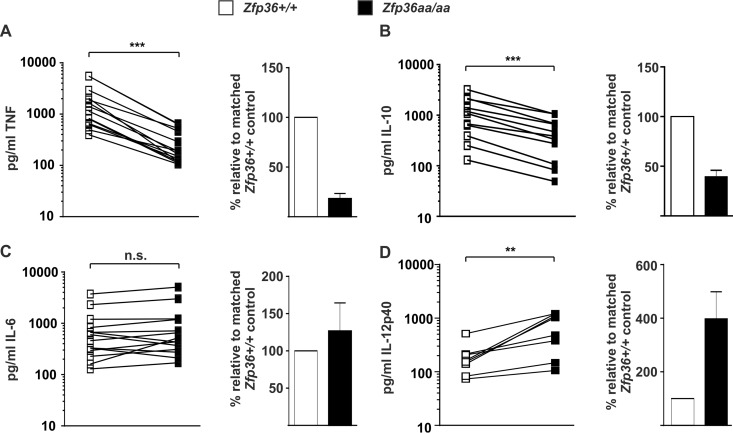
Expression of TNF, IL-10, IL-6, and IL-12p40 proteins by *Zfp36*^+/+^ and *Zfp36*^*aa/aa*^ M-BMMs. Matched *Zfp36*^+/+^ and *Zfp36*^*aa/aa*^ M-BMMs were cultured in 24-, 12-, or 6-well dishes and stimulated with 10 ng/ml LPS for 4 h. ELISAs or multiplex bead assays were used to measure TNF (A), IL-10 (B), IL-6 (C), and IL-12p40 (D) levels. In the cases of TNF and IL-6, 5 of 14 experiments employed matched *Zfp36*^+/+^ and *Zfp36*^*aa/aa*^ littermates. All other experiments employed at least three mice of each genotype, with mean cytokine concentrations being plotted. Cytokine quantities expressed by matched *Zfp36*^+/+^ and *Zfp36*^*aa/aa*^ M-BMMs or sets of M-BMMs are connected by lines. The graphs on the right show mean cytokine expression levels in *Zfp36*^*aa/aa*^ M-BMMs relative to those in matched *Zfp36*^+/+^ controls. n.s., not statistically significant; ***, *P* < 0.005; **, *P* < 0.01 (by a Wilcoxon matched-pair test).

TTP can negatively regulate gene expression by inhibiting translation rather than destabilizing mRNA (37, 57, 58). The LPS-induced expression levels of the TNF, IL-6, IL-10, and IL-12p40 proteins between *Zfp36*^+/+^ and *Zfp36*^*aa/aa*^ M-BMMs were therefore compared. Multiple experiments were performed by using different numbers and densities of M-BMMs, depending on whether the primary aim was to harvest RNA for microarrays, RNA for quantitative PCR (qPCR), secreted protein for enzyme-linked immunosorbent assays (ELISAs) and multiplex assays, or intracellular protein for Western blotting. Regardless of the experimental conditions, the expression levels of both the TNF and IL-10 proteins were consistently diminished in *Zfp36*^*aa/aa*^ M-BMMs (by approximately 5-fold and 3-fold, respectively) (Fig. 2A and B). In contrast, the pattern of expression of IL-6 was inconsistent, with unchanged expression by *Zfp36*^*aa/aa*^ M-BMMs in most cases but both overexpression and underexpression in some experiments. Overall, there was no statistically significant difference in the expression levels of IL-6 between *Zfp36*^+/+^ and *Zfp36*^*aa/aa*^ M-BMMs. (Fig. 2C). The expression level of the IL-12p40 protein was invariably higher in *Zfp36*^*aa/aa*^ than in matched *Zfp36*^+/+^ M-BMMs, although the extent of overexpression was quite variable (Fig. 2D).

### Anomalous expression of certain TTP targets by *Dusp1*^−/−^ macrophages.

**FIG 3:**
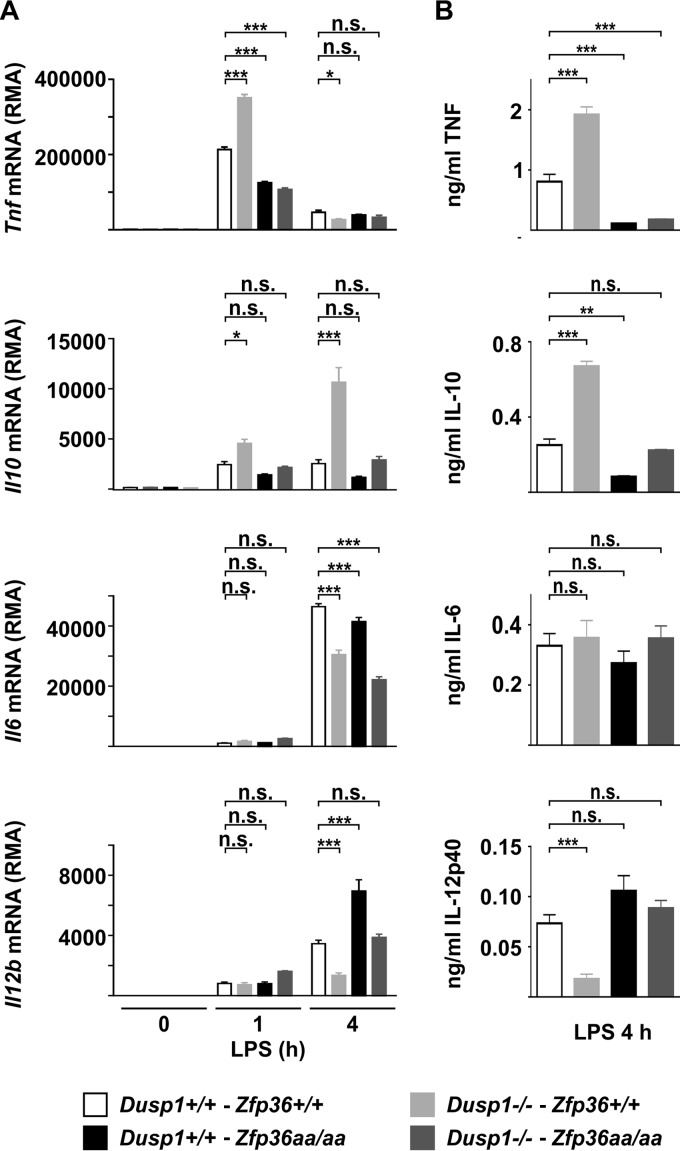
Regulation of inflammatory mediators by DUSP1 and TTP. Wild-type, *Dusp1*^−/−^, *Zfp36*^*aa/aa*^, and *Dusp1*^−/−^-*Zfp36*^*aa/aa*^ M-BMMs were stimulated with 10 ng/ml LPS for 1 h or 4 h. Expression levels of selected mRNAs were determined by microarray analysis (A), and the levels of the corresponding proteins were measured by an ELISA or a multiplex bead assay (B). Graphs represent means ± standard errors of the means of data from 3 independent M-BMM cultures. n.s., not statistically significant; *, *P* < 0.05; **, *P* < 0.01; ***, *P* < 0.005 (by one-way ANOVA). The microarray experiment used to generate the data in panel A was previously described (52).

In *Dusp1*^−/−^ macrophages, enhanced and prolonged MAPK p38 activation in response to LPS promotes the overexpression of several cytokines and chemokines via increased phosphorylation and inactivation of TTP (52). The defining characteristic of genes that are regulated by the DUSP1-TTP axis is that the overexpression caused by the disruption of the *Dusp1* gene can be prevented by combining this with the *Zfp36*^*aa/aa*^ genotype. Dysregulated MAPK p38 signaling fails to promote the overexpression of target genes if TTP cannot be phosphorylated and inactivated. At the protein and mRNA levels, the expression levels of both TNF and the anti-inflammatory cytokine IL-10 conformed to the pattern described above, being elevated in *Dusp1*^−/−^ but not *Dusp1*^−/−^-*Zfp36*^*aa/aa*^ M-BMMs (Fig. 3). *Il6* mRNA levels were decreased in M-BMMs of all three genetically modified genotypes, but there were no significant differences in protein expression levels (Fig. 3). At 4 h, the expression level of *Il12b* mRNA was actually decreased rather than increased in *Dusp1*^−/−^ M-BMMs and was increased rather than decreased in *Zfp36*^*aa/aa*^ M-BMMs, whereas in *Dusp1*^−/−^-*Zfp36*^*aa/aa*^ M-BMMs, the expression level of *Il12b* mRNA was not significantly different from that in wild-type controls (Fig. 3). The pattern of expression of the IL-12p40 protein was similar.

### Targeted mutation of TTP paradoxically increases transcription of the *Il6* and *Il12b* genes.

**FIG 4:**
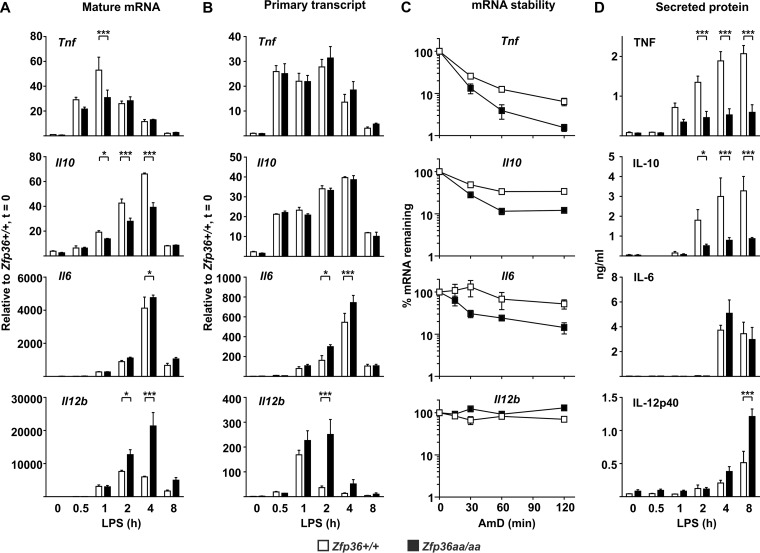
TTP mutation affects gene expression at different levels. *Zfp36*^+/+^ and *Zfp36*^*aa/aa*^ M-BMMs were treated with LPS for the times indicated, supernatants were collected, and RNA was isolated. (A) Steady-state mRNA abundance was measured by quantitative PCR. (B) Levels of primary transcripts were measured by quantitative PCR. (C) Actinomycin D (AmD) chase experiments were performed at the peak of gene expression (1 h in the case of *Tnf* and 4 h in the cases of *Il10*, *Il6*, and *Il12b*). (D) Levels of secreted proteins were measured by an ELISA or a multiplex bead assay. Graphs represent means ± standard errors of the means of data from three independent M-BMM cultures in each case. Pairwise comparisons that showed no statistical significance are not indicated. *, *P* < 0.05; **, *P* < 0.01; ***, *P* < 0.005 (by a Mann-Whitney test).

*Tnf*, *Il6*, *Il10*, and *Il12b* mRNAs can all be physically recognized by TTP (38) yet show very different responses to mutations that influence TTP phosphorylation and activity. To make sense of these disparate patterns, we monitored gene expression at the levels of steady-state mRNA, primary transcripts (as a surrogate for the transcription rate), mRNA stability, and protein secretion in LPS-treated *Zfp36*^+/+^ and *Zfp36*^*aa/aa*^ M-BMMs (Fig. 4).

The abundances of both *Tnf* and *Il10* steady-state mRNAs were decreased in *Zfp36*^*aa/aa*^ M-BMMs (Fig. 4A). The expression levels of the corresponding proteins were diminished (Fig. 4D), as previously shown (53) (Fig. 2). There were no significant differences in *Tnf* or *Il10* primary transcript levels (Fig. 4B), but the stabilities of *Tnf* and *Il10* mRNAs were decreased in *Zfp36*^*aa/aa*^ M-BMMs (Fig. 4C), as previously reported (53). The behavior of both the *Tnf* and *Il10* genes is therefore consistent with direct regulation by TTP at the level of mRNA stability. Since the *Zfp36* gene mutation had a greater effect on the TNF and IL-10 proteins than on *Tnf* and *Il10* mRNAs, the phosphorylation of TTP may also have some impact at the translational level, as was previously suggested (37, 57, 58).

Only a minor difference in the steady-state *Il6* mRNA abundance was detected at 4 h (Fig. 4A), and IL-6 secretion did not differ between *Zfp36*^+/+^ and *Zfp36*^*aa/aa*^ M-BMMs (Fig. 4D). There appeared to be an almost perfect balance between the increase in the rate of *Il6* transcription (Fig. 4B) and the increase in the rate of *Il6* mRNA degradation (Fig. 4C). *Il12b* mRNA was highly stable in both *Zfp36*^+/+^ and *Zfp36*^*aa/aa*^ M-BMMs (Fig. 4C). The increased expression levels of *Il12b* mRNA (Fig. 4A) and IL-12p40 protein (Fig. 4D) by *Zfp36*^*aa/aa*^ M-BMMs appeared to be driven by an enhanced transcriptional response to LPS (Fig. 4B).

### Disruption of IL-10-mediated negative feedback contributes to the anomalous expression of IL-6 and IL-12p40 by *Zfp36*^*aa/aa*^ macrophages.

**FIG 5:**
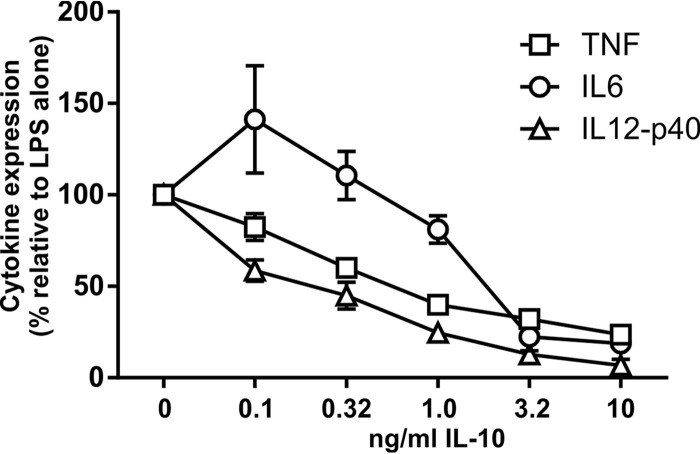
Dose-dependent inhibition of proinflammatory gene expression by IL-10. *Il10*^−/−^ M-BMMs were stimulated with 10 ng/ml LPS for 4 h in the presence of different concentrations of recombinant IL-10. TNF, IL-6, and IL-12p40 levels were quantified by an ELISA. Cytokine levels were normalized against those in the absence of IL-10. The graph shows means ± standard errors of the means of data from at least 8 independent M-BMM cultures.

IL-10 is a well-characterized target of TTP (38–41), which is underexpressed by *Zfp36*^*aa/aa*^ M-BMMs (Fig. 5) and known to negatively regulate the expression of TNF, IL-12p40, and IL-6 (7, 19). Noticeably, the expression of IL-12p40 in macrophages of differing genetic backgrounds was an almost perfect mirror image of IL-10 expression (Fig. 3). It was therefore speculated that a disruption of IL-10-mediated autocrine negative feedback mechanisms contributed to differences in IL-6 and IL-12p40 expression *in vitro*.

It is not known whether proinflammatory genes display the same dose response to inhibition by IL-10. Differential sensitivity to IL-10 could influence the outcomes of dysregulated IL-10 biosynthesis in macrophages. Therefore, *Il10*^−/−^ M-BMMs were used to investigate the relative sensitivity of TNF, IL-12p40, and IL-6 expression to exogenous IL-10 in the absence of interference from endogenous IL-10 (Fig. 5). The three proinflammatory cytokines displayed quite distinct dose responses. IL-12p40 was the most sensitive in terms of both the 50% effective concentration (EC_50_) (estimated to be 0.15 ng/ml) and maximal inhibition (93%). TNF was inhibited by a maximum of 76% and with an EC_50_ of approximately 0.29 ng/ml. IL-6 biosynthesis was insensitive to low concentrations of IL-10. Maximal inhibition was 81%, and the estimated EC_50_ was 1.52 ng/ml.

**FIG 6:**
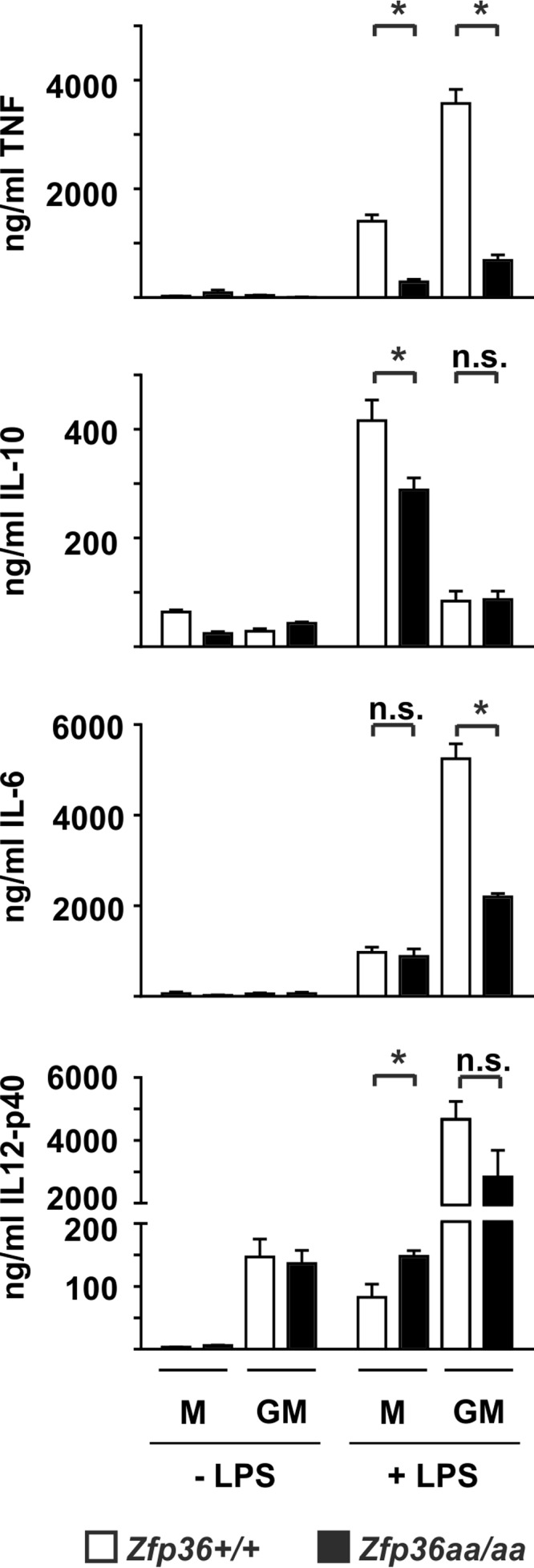
Differential gene expression in *Zfp36*^+/+^ and *Zfp36*^*aa/aa*^ M-BMMs and GM-BMMs. Macrophages were differentiated from bone marrow of *Zfp36*^+/+^ and *Zfp36*^*aa/aa*^ mice in the presence of either M-CSF of GM-CSF and treated with 10 ng/ml LPS for 4 h. Cytokine levels were measured by an ELISA or a multiplex bead assay. Graphs represent means ± standard errors of the means from 4 independent cultures (TNF, IL-10, and IL-12p40) or 7 independent cultures (IL-6). n.s., not statistically significant; *, *P* < 0.05 (by a Mann-Whitney test).

Differentiation of BMMs in the presence of granulocyte-macrophage colony-stimulating factor (GM-CSF) rather than M-CSF has been reported to generate a cell population (GM-BMMs) that expresses relatively little IL-10 but larger amounts of TNF, IL-6, and IL-12p40 in response to LPS (59, 60). It was confirmed that wild-type GM-BMMs expressed 5-fold less IL-10 than did M-BMMs generated in parallel from the same donor mice (Fig. 6). The expression levels of the proinflammatory cytokines TNF, IL-6, and IL-12p40 were elevated 2.5-fold, 5.4-fold, and 56.3-fold, respectively, in GM-BMMs compared to M-BMMs generated in parallel. *Zfp36*^+/+^ and *Zfp36*^*aa/aa*^ GM-BMMs did not differ in their expression of IL-10 after stimulation with LPS. Therefore, in GM-BMMs, the targeted mutation of TTP cannot indirectly influence gene expression by perturbing IL-10-mediated autocrine/paracrine feedback mechanisms. Targeted mutation of the *Zfp36* locus decreased the expression of TNF in both M-BMMs and GM-BMMs. The *Zfp36* mutation did not affect IL-6 expression in M-BMMs, whereas in GM-BMMs, the mutation significantly decreased the expression of IL-6. The expression of IL-12p40 was elevated as a consequence of the *Zfp36* mutation in M-BMMs. In contrast, in GM-BMMs, the *Zfp36* mutation had no significant effect on IL-12p40 expression.

**FIG 7:**
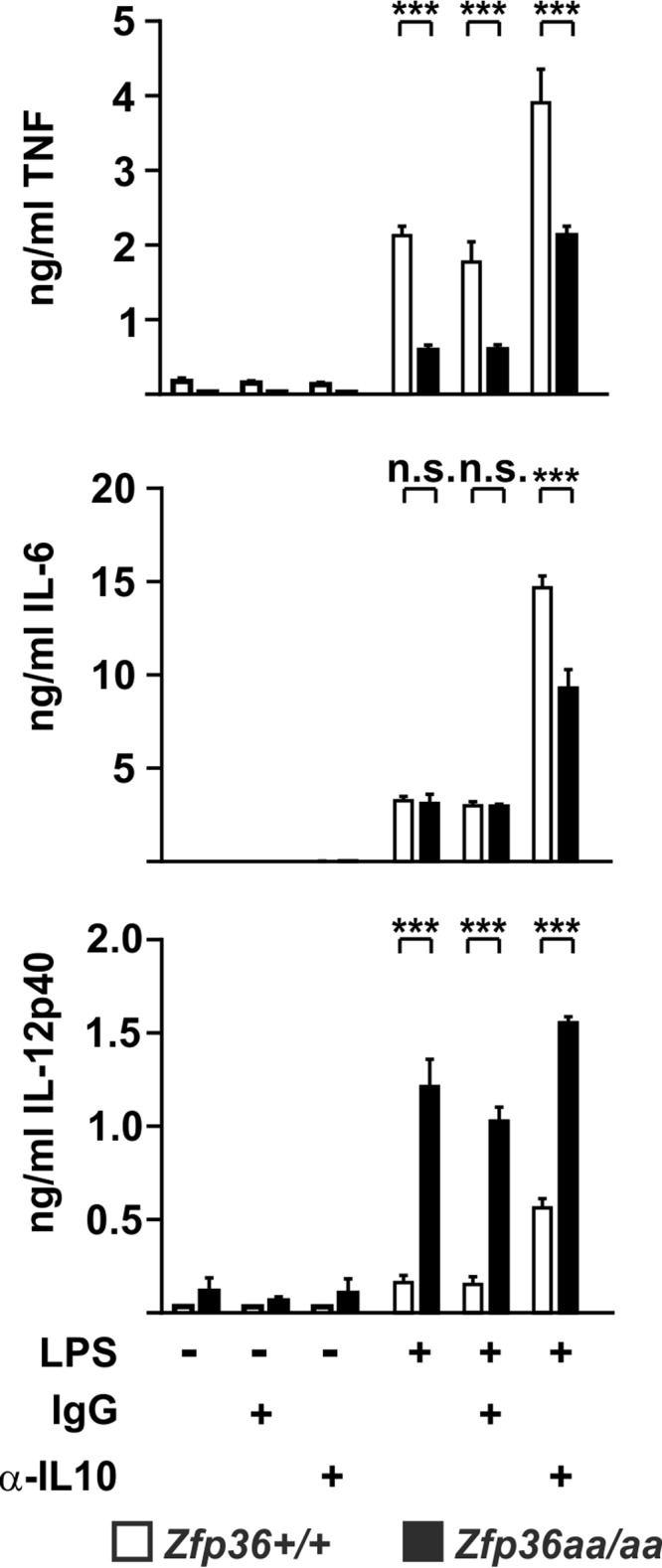
Endogenous IL-10 differentially affects the expression of proinflammatory cytokines. *Zfp36*^+/+^ and *Zfp36*^*aa/aa*^ M-BMMs were treated with 10 ng/ml LPS for 4 h (TNF and IL-6) or 8 h (IL-12p40) in the presence of 10 μg/ml IL-10-neutralizing antibody or an isotype control. Cytokine levels were measured by a multiplex bead assay or an ELISA. Graphs represent means ± standard errors of the means from 4 independent M-BMM cultures. n.s., not statistically significant; ***, *P* < 0.005 (by a Mann-Whitney test).

The observations described above support the hypothesis that changes in the expression of IL-10 influence the phenotype caused by the substitution of two TTP phosphorylation sites. To test this hypothesis more rigorously, *Zfp36*^+/+^ and *Zfp36*^*aa/aa*^ M-BMMs were stimulated with LPS in the presence of an IL-10-neutralizing antibody or an isotype control (Fig. 7). In the presence of the neutralizing antibody, IL-6 was underexpressed by *Zfp36*^*aa/aa*^ M-BMMs. IL-10 neutralization increased the expression of IL-12p40 in *Zfp36*^+/+^ M-BMMs but had relatively little effect on *Zfp36*^*aa/aa*^ M-BMMs. As a consequence, the degree of overexpression of IL-12p40 by *Zfp36*^*aa/aa*^ M-BMMs was diminished. Together, these data indicate that the aberrant patterns of expression of IL-6 (unaltered in *Zfp36*^*aa/aa*^ M-BMMs) and IL-12p40 (elevated in *Zfp36*^*aa/aa*^ M-BMMs) are at least partly due to alterations in IL-10-mediated negative feedback. The expression of TNF appears minimally sensitive to this phenomenon.

### Targeted mutation of TTP has overwhelmingly anti-inflammatory consequences *in vivo*.

**FIG 8:**
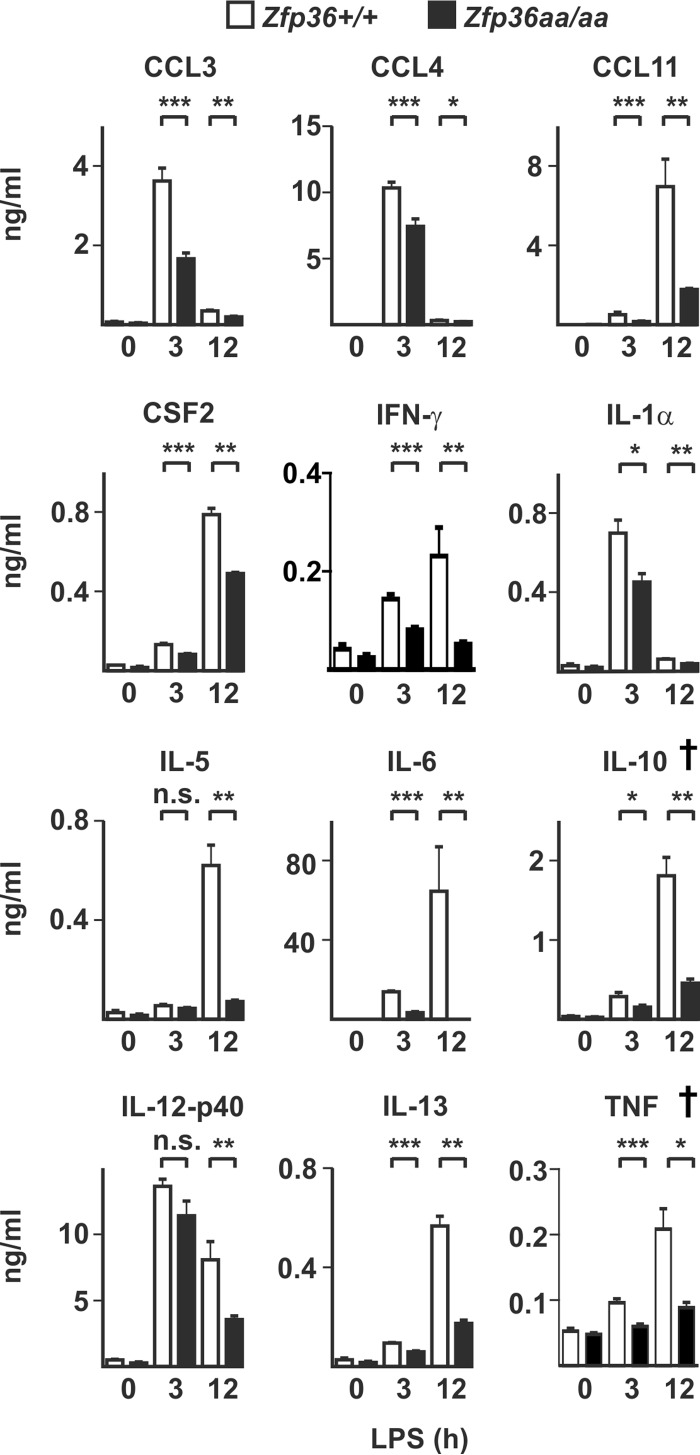
*In vivo* responses to LPS are broadly impaired in *Zfp36*^*aa/aa*^ mice. *Zfp36*^+/+^ and *Zfp36*^*aa/aa*^ mice were injected intraperitoneally with 5 mg/kg LPS and humanely sacrificed after 3 or 12 h. Serum cytokine levels were measured by a multiplex bead assay or an ELISA. Graphs represent means ± standard errors of the means for 5 untreated mice and 10 LPS-injected mice at each time point. *, *P* < 0.05; **, *P* < 0.01; ***, *P* < 0.005 (by a Mann-Whitney test). The IL-10 and TNF measurements (indicated by †) were previously reported (53).

Next, *Zfp36*^+/+^ and *Zfp36*^*aa/aa*^ mice were injected intraperitoneally (i.p.) with LPS, and serum cytokine and chemokine levels were measured after 3 and 12 h (Fig. 8). Decreased expression levels of TNF and IL-10 in *Zfp36*^*aa/aa*^ mice were previously reported (53) (indicated by † in Fig. 8). CCL3, CCL4, CCL11, CSF2, gamma interferon (IFN-γ), IL-1α, IL-5, and IL-13 were also significantly underexpressed by *Zfp36*^*aa/aa*^ mice. Most notably, the expression level of IL-6 was decreased by almost 4-fold at 3 h and by more than 200-fold at 12 h in *Zfp36*^*aa/aa*^ mice. The elevated expression of IL-12p40 in LPS-treated *Zfp36*^*aa/aa*^ M-BMMs was not recapitulated *in vivo*. Serum concentrations of IL-12p40 were not significantly different between LPS-treated *Zfp36*^+/+^ and *Zfp36*^*aa/aa*^ mice at 3 h but were significantly lower in *Zfp36*^*aa/aa*^ mice at 12 h.

**FIG 9:**
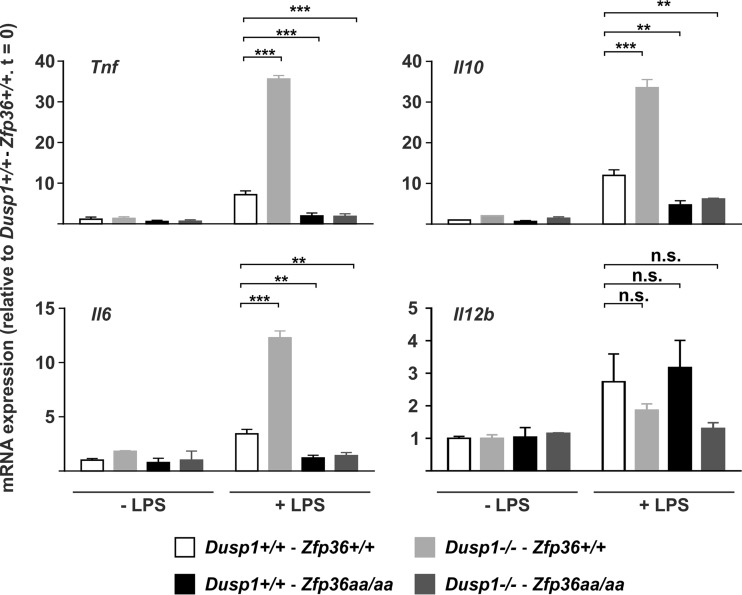
Regulation of cytokine expression by the DUSP1-TTP axis *in vivo. Dusp1*^+/+^-*Zfp36*^+/+^, *Dusp1*^−/−^-*Zfp36*^+/+^, *Dusp1*^+/+^-*Zfp36*^*aa/aa*^, or *Dusp1*^−/−^-*Zfp36*^*aa/aa*^ mice were injected intraperitoneally with 5 mg/kg LPS and humanely sacrificed after 3 h. Spleens were excised, RNA was prepared, and the levels of the indicated transcripts were quantified by qPCR, with normalization against *B2m* mRNA levels and then against the untreated wild-type (*Dusp1*^+/+^-*Zfp36*^+/+^) control. Graphs represent means ± standard errors of the means for 2 untreated and 4 LPS-treated mice of each genotype. n.s., not statistically significant; **, *P* < 0.01; ***, *P* < 0.005 (by ANOVA).

Finally, to test whether cytokine expression *in vivo* was regulated by DUSP1 via alterations in TTP phosphorylation, wild-type, *Dusp1*^−/−^, *Zfp36*^*aa/aa*^, and *Dusp1*^−/−^-*Zfp36aa/aa* mice were injected intraperitoneally with LPS and culled after 3 h, and the expression levels of *Tnf*, *Il10*, *Il6*, and *IL12b* mRNAs were measured in spleens (Fig. 9). Levels of *Tnf* and *Il10* mRNAs were elevated in spleens of LPS-treated *Dusp1*^−/−^ mice and decreased in those of *Zfp36*^*aa/aa*^ mice. When *Dusp1*^−/−^ and *Zfp36*^*aa/aa*^ genetic modifications were combined, the *Zfp36*^*aa/aa*^ phenotype was dominant, and the expression levels of both mRNAs remained low. These patterns of gene expression are very similar to those previously described *in vitro* (Fig. 3A) and in serum of LPS-treated mice (52), consistent with DUSP1 regulating gene expression via modulation of the phosphorylation state of TTP in both contexts. Strikingly, the pattern of expression of *Il6* mRNA was the same: it was increased in *Dusp1*^−/−^ spleens but decreased in both *Zfp36*^*aa/aa*^ and *Dusp1*^−/−^-*Zfp36*^*aa/aa*^ spleens, indicating a similar mechanism of regulation. The level of the IL-6 protein was also very strongly elevated in sera of LPS-treated *Dusp1*^−/−^ mice but decreased in sera of both *Zfp36*^*aa/aa*^ and *Dusp1*^−/−^-*Zfp36*^*aa/aa*^ mice (data not shown). Differences in the expression levels of *Il12b* mRNA among the four genotypes of mice were not significant. The dysregulation of *Il12b* gene expression in isolated *Zfp36*^*aa/aa*^ M-BMMs was therefore not recapitulated *in vivo* in *Zfp36*^*aa/aa*^ mice (Fig. 8).

## DISCUSSION

The phosphorylation and dephosphorylation of TTP constitute a molecular switch for turning gene expression on and off during responses to inflammatory stimuli (43). In *Dusp1*^−/−^ cells, the dysregulation of MAPK p38 signaling pushes the switch in the “on” direction, promoting the phosphorylation and inactivation of TTP and enhancing and sustaining the expression of TTP target genes by increasing mRNA stability. In *Zfp36*^*aa/aa*^ cells, the switch is locked in the “off” position, and the expression of TTP target genes is diminished as a consequence of enhanced mRNA decay. This locked switch prevents the overexpression of TTP target genes in cells in which the two genetic modifications are combined (43, 52). Here and in previous reports (52, 53), the expression of both TNF and IL-10 conformed exactly to the pattern described above, whether investigated *in vitro* or *in vivo*. The behavior of these genes can be understood simply in terms of our working model of TTP function. However, it remains puzzling at first sight that the same molecular mechanism is used to regulate the expressions of a powerful proinflammatory cytokine and a powerful anti-inflammatory cytokine. It is likely that this shared regulation contributes to the resolution of inflammation. Any stimulus that provokes the strong expression of TNF via the activation of the MAPK p38 and the phosphorylation of TTP will also provoke the strong expression of IL-10, with a built-in delay owing to the slower transcriptional activation of the latter. IL-10 then acts as an autocrine or paracrine negative feedback regulator of the inflammatory response.

When gene expression was initially studied in *Zfp36*^*aa/aa*^ M-BMMs, we were puzzled that IL-6 did not behave as expected. IL-6 has been well characterized as a target of TTP (30, 55, 56), yet its expression was not consistently diminished in *Zfp36*^*aa/aa*^ M-BMMs and in many experiments was actually increased (Fig. 2). Microarray analysis also revealed the surprising overexpression of several transcripts by *Zfp36*^*aa/aa*^ M-BMMs (Fig. 1), among which *Il12b* was prominent. We hypothesized that an impairment of IL-10-mediated negative feedback control contributed to these anomalies. Being a secondary response gene, the peak of expression of *Il6* occurred at a time when the endogenous IL-10 protein had already accumulated in tissue culture supernatants (Fig. 4). IL-6 expression was sensitive to inhibition by IL-10, with a very steep dose response at concentrations of between 1 and 3 ng/ml (Fig. 5), similar to the range of IL-10 concentrations produced by *Zfp36*^+/+^ and *Zfp36*^*aa/aa*^ M-BMMs (Fig. 4). As predicted, IL-6 was underexpressed by *Zfp36*^*aa/aa*^ GM-BMMs (Fig. 6) and by *Zfp36*^*aa/aa*^ peritoneal macrophages (data not shown), both of which produce very low levels of IL-10. Neutralization of IL-10 also restored the expected gene expression pattern, in which IL-6 was underexpressed by *Zfp36*^*aa/aa*^ M-BMMs (Fig. 7). It therefore appears that differences in the levels of IL-10 in tissue culture have a profound effect on the expression of the *Il6* gene, resulting in variable under- or overexpression by *Zfp36*^*aa/aa*^ M-BMMs. Although *Tnf* gene expression is also negatively regulated by IL-10 (Fig. 5) (19, 61), its immediate early pattern of expression (61, 62) (Fig. 4) presumably allows it to escape the influence of such fluctuations in the efficacy of negative feedback control. By the time that levels of endogenous IL-10 accumulate significantly, the transcriptional activity of the *Tnf* gene is already declining.

The production of the IL-12p40 protein was profoundly sensitive to IL-10-mediated inhibition (Fig. 5). The paradoxical overexpression of IL-12p40 by *Zfp36*^*aa/aa*^ M-BMMs was caused by an increase in transcription (Fig. 4). Like others (63, 64), we found no evidence that TTP regulated *Il12b* mRNA stability (Fig. 4). This increase of expression was not observed in *Zfp36*^*aa/aa*^ GM-BMMs, which express little IL-10 (Fig. 6). IL-10 has been reported to inhibit *Il12b* transcription via the induction of the transcriptional repressor NFIL3 (nuclear factor, IL-3 regulated) (13). Four hours after the addition of LPS, the expression level of *Nfil3* mRNA was significantly lower in *Zfp36*^*aa/aa*^ than in *Zfp36*^+/+^ M-BMMs (2,135 ± 35 versus 4,940 ± 497 robust microarray analysis units [RMA]; *P* < 0.005). It is therefore possible that impaired negative feedback via NFIL3 contributes to the enhanced transcriptional induction of the *Il12b* gene in *Zfp36*^*aa/aa*^ M-BMMs (Fig. 4). However, neutralization of IL-10 only partially reversed the overexpression of IL-12p40 by *Zfp36*^*aa/aa*^ M-BMMs, suggesting that additional mechanisms may be involved. TTP has been reported to inhibit *Il12b* gene expression by modulating the function of the transcription factor NF-κB (nuclear factor kappa light chain enhancer of activated B cells) (65). However, we were unable to detect any differences in NF-κB function in *Zfp36*^*aa/aa*^ M-BMMs (53). Additionally, it is difficult to explain the increases in the expression levels of only a few genes by the increased activity of a transcription factor that regulates much of the macrophage response to LPS. Therefore, the aberrant expression of IL-12p40 by *Zfp36*^*aa/aa*^ M-BMMs has been only partially accounted for.

Previous reports identified IL-10 as a target of TTP (39, 41), describing the increased expression of IL-10 and the consequent underexpression of both IL-6 and IL-12 by *Zfp36*^−/−^ macrophages. The phenomenon described here is a mirror image, in which a gain of function of TTP impairs IL-10-mediated negative feedback control *in vitro* and promotes the increased expressions of these two cytokines. As we and others are interested in the concept of therapeutic targeting of TTP (31, 43, 53), the impairment of negative feedback control was a source of concern, prompting us to test the *in vivo* effects of the gain-of-function mutation. Despite the fact that LPS-induced IL-10 expression was diminished in *Zfp36*^*aa/aa*^ mice, we detected no corresponding increase in the expression of either IL-6 or IL-12p40 (Fig. 8 and 9). In fact, IL-6 was consistently expressed at very low levels by *Zfp36*^*aa/aa*^ mice following systemic LPS challenge (Fig. 8), the injection of zymosan into dorsal air pouches, or the induction of experimental arthritis (53). Increased circulating levels of either IL-12 or IL-23, which share the common IL-12p40 subunit, would be expected to promote Th1 and Th17 responses, characterized by elevated expression levels of IFN-γ and IL-17. In fact, both of these effector cytokines were present at lower levels in sera of LPS-injected *Zfp36*^*aa/aa*^ mice (49) (Fig. 8).These findings suggest that agonists that activate TTP may exert anti-inflammatory effects without incurring immune activation via the increased expression of IL-6, IL-12, or IL-23. It will be important to test this further in experimental settings such as inflammatory bowel disease, where impaired expression of IL-10 may have profound consequences (8, 12).

A remaining question is why the behaviors of the *Il6* and *Il12b* genes are so different when studied *in vitro* and *in vivo*. Although *in vitro* experiments are invaluable for gaining insight into molecular mechanisms of gene regulation, it is easy to forget how poorly they model inflammatory responses *in vivo*. In many senses, the *in vitro* assay system is artificial. The population of cells is homogeneous, their exposure to stimulus is synchronous, and they remain thereafter in a static milieu, in which local concentrations of cytokines and other effectors may become high and autocrine or paracrine effects may be amplified. *In vivo*, the population of cells responding to LPS is heterogeneous, their time of exposure to the agonist will vary somewhat, and the local accumulation of cytokines and other immune effectors will be limited by circulation. Whatever the explanation for the divergent behaviors of IL-6 and IL-12p40 *in vitro* and *in vivo*, our principal conclusion remains the same. A gain of function of TTP causes the dysregulation of IL-6 and IL-12p40, at least in part indirectly, via changes in IL-10 expression. However, aberrant overexpression of IL-6 and IL-12p40 does not occur in *Zfp36*^*aa/aa*^ mice.

## MATERIALS AND METHODS

### Materials.

LPS (Escherichia coli serotype EH100) was purchased from Enzo Life Sciences. Other biochemicals were purchased from Sigma-Aldrich unless otherwise stated. All media and sera were routinely tested for endotoxin by using the Limulus amebocyte lysate test (Lonza) and were rejected if the endotoxin concentration exceeded 0.1 U/ml.

### Generation of mouse strains.

The generation of the *Zfp36*^*aa/aa*^ mouse strain in the C57BL/6 background was described previously (49). The *Dusp1*^−/−^ strain was a generous gift from Bristol-Myers Squibb and was backcrossed to the C57BL/6 strain for 10 generations prior to performing the experiments described here. The double-targeted *Dusp1*^−/−^-*Zfp36*^*aa/aa*^ line was generated by three generations of crossing, with genotyping being performed by PCR of genomic DNA, and thereafter was maintained as a pure-breeding line. Bone marrow from *Il10*^−/−^ mice was generously provided by Fiona Powrie (Oxford).

### *In vivo* experiments and cell isolation.

All animal experiments were approved by local ethical committees and performed under UK Home Office project licenses. C57BL/6 mice were purchased from Harlan Laboratories. All mice used were between 6 and 12 weeks of age. To assess the systemic response to LPS, mice were injected i.p. with 5 mg/kg of body weight of purified LPS in 200 μl sterile phosphate-buffered saline (PBS). Mice were humanely culled 3 h or 12 h after challenge, and peripheral blood was collected by cardiac puncture for serum isolation. Spleens were excised and snap-frozen in liquid nitrogen for later isolation of RNA.

Bone marrow was isolated from humanely culled mice, and BMMs (bone marrow-derived macrophages) were obtained by differentiation *in vitro* with either 100 ng/ml M-CSF (Peprotech), or 50 ng/ml GM-CSF in RPMI 1640 containing 10% heat-inactivated fetal calf serum (FCS) and penicillin-streptomycin for 7 days. BMMs were plated at a density of 1 × 10^6^ macrophages/ml in the appropriate cell culture plate at least 1 day prior to stimulation.

Neutralization of endogenous secreted IL-10 was achieved by *in vitro* incubation with an IL-10-neutralizing antibody (catalogue number 501407; BioLegend) at 10 μg/ml for the duration of the assays. Controls were treated with an equal concentration of isotype-matched antibody.

### Measurements of mRNA levels.

RNA was extracted from BMMs by using QIAshredder columns and an RNeasy minikit (Qiagen). cDNA was generated by using the iScript cDNA synthesis kit (Bio-Rad). Gene expression was quantified by reverse transcription-quantitative PCR (qRT-PCR) on a LightCycler 480 II instrument (Roche) using the Superscript III platinum RT-PCR kit and custom-synthesized oligonucleotide primers (Eurofins MWG) with SYBR Premix Ex *Taq* (Lonza). The relative gene expression level was calculated by using the ΔΔ*C_T_* method with *Gapdh* or *B2m* mRNA for the normalization of RNA levels. Primary transcript PCR was performed by using primer pairs that crossed exon-intron boundaries, with an additional DNase I step to remove contaminating genomic DNA from RNA samples (Qiagen). Control PCRs were carried out in the absence of RT in order to monitor genomic DNA contamination, which in all cases was negligible. Sequences of oligonucleotides designed to detect primary or mature transcripts are available from the authors upon request.

The half-life of mRNA was estimated by using the transcription inhibitor actinomycin D (Sigma) at 5 μg/ml, followed by measurement of mRNA levels by quantitative real-time PCR.

Microarray analyses were performed by using SurePrint G3 Mouse GE 8x60K slides (Agilent) and Partek Genomics Suite version 6.6, build 6.13.0315 (Partek), as previously described (49). For the generation of volcano plots, transcripts were first filtered for significant upregulation in response to LPS (>2-fold increase; adjusted *P* value of <0.05), and weakly expressed transcripts were removed by the application of an arbitrary filter of 200 RMA. Plots (log_2_ fold difference of expression versus −log_10_ analysis of variance [ANOVA] *P* value) were constructed by using ggplot2s in the R statistical package, with subset cutoffs at a *P* value of <0.05 and a fold difference of expression of >1.5.

### Assessment of protein expression.

Secreted factors in tissue culture supernatants and sera were quantified by an ELISA according to the manufacturer's instructions (eBioscience) or by using Bio-Plex bead capture assays and a Bio-Plex 200 analyzer (Bio-Rad).

### Statistical analysis.

GraphPad Prism software (version 5.03) was used for statistical analysis. A Mann-Whitney U test was used for comparisons of two groups. For analysis of multiple groups, ANOVA with Bonferroni correction for multiple comparisons was used.

### Accession number.

Microarray data discussed in this paper were deposited at the Gene Expression Omnibus (http://www.ncbi.nlm.nih.gov/geo/) with accession number GSE68449.

## Supplementary Material

Supplemental material
