# Supplementary material for: Gain-of-Function Mutation of Tristetraprolin Impairs Negative Feedback Control of Macrophages In Vitro yet Has Overwhelmingly Anti-Inflammatory Consequences In Vivo
Source: Mol Cell Biol. 2017 May 16;37(11):e00536-16. doi: 10.1128/MCB.00536-16 (PMC5440651; doi:10.1128/MCB.00536-16)
Supplement: Supplemental material [file supp_37_11_e00536-16__index.html]

Supplemental material 

# Gain-of-Function Mutation of Tristetraprolin Impairs Negative Feedback Control of Macrophages *In Vitro* yet Has Overwhelmingly Anti-Inflammatory Consequences *In Vivo*

## Supplemental material

- Supplemental file 1 -

  Table S1 (Differential gene expression in Zfp36aa/aa M-BMMs)

  XLSX, 218K
- Supplemental file 2 -

  Table S2 (GO analysis of differentially expressed genes in Zfp36aa/aa M-BMMs)

  XLSX, 4.2M
